# Clinical and Hematological Relevance of *JAK2*V617F, *CALR*, and *MPL* Mutations in Vietnamese Patients with Essential Thrombocythemia

**DOI:** 10.31557/APJCP.2019.20.9.2775

**Published:** 2019

**Authors:** Hoang Anh Vu, Tran Thi Thao, Cao Van Dong, Nguyen Lam Vuong, Ho Quoc Chuong, Phan Nguyen Thanh Van, Huynh Nghia, Nguyen Tan Binh, Phu Chi Dung, Phan Thi Xinh

**Affiliations:** 1 *Center for Molecular Biomedicine,*; 2 *Department of Hematology, Faculty of Medicine,*; 4 *Department of Medical Statistics and Informatics, Faculty of Public Health, University of Medicine and Pharmacy at Ho Chi Minh City,*; 3 *Ho Chi Minh City Blood Transfusion and Hematology Hospital, Ho Chi Minh City, Vietnam.*

**Keywords:** Essential thrombocythemia, JAK2V617F, CALR, MPL, Vietnam

## Abstract

**Background:**

The picture of Vietnamese patients with essential thrombocythemia (ET) remains mostly undetermined. Our study intended to determine the frequency of *JAK2*V617F, *CALR* exon 9, and *MPL* exon 10 mutations as well as to analyze clinical characteristics associated with different mutational status in Vietnamese ET patients.

**Methods:**

We explored mutations of *JAK2*V617F, *MPL*, and *CALR* from 395 patients using allele specific oligonucleotide – polymerase chain reaction and Sanger sequencing techniques; then, the clinical and hematological features were compared according to mutation patterns.

**Results:**

We found that *JAK2*V617F, *CALR* exon 9, and *MPL* exon 10 mutations were present in 56.2%, 27.6%, and 1% of the 395 patients with ET, respectively. Twelve different types of *CALR* mutation were detected in 109 patients, with the *CALR* type 1 mutation (c.1099_1150del; L367fs*46) was the most common, followed by *CALR* type 2 mutation (c.1154_1155insTTGTC; K385fs*47). The *JAK2*V617F-positive patients had older age, higher white blood cell counts and higher hemoglobin levels but lower platelet counts than patients with *CALR* mutations or patients negative for triple tests. There was no significant difference regarding sex ratio, white blood cell counts, platelet counts and hemoglobin levels among *CALR* mutation subtypes.

**Conclusion:**

we reported high frequency of *JAK2*V617F, *CALR*, and *MPL* mutations in Vietnamese patients with ET and underscored the importance of combined genetic tests for diagnosis and classification of ET into different subtypes.

## Introduction

Essential thrombocythemia (ET), a subtype of the *BCR-ABL1*-negative myeloproliferative neoplasms (MPNs), is a clonal hematopoietic stem cell disorder characterized by an isolated thrombocytosis and associated with complications such as thrombosis, hemorrhage, and progression to myelofibrosis or acute myeloid leukemia. The three most common *BCR-ABL1*-negative MPNs are polycythemia vera (PV), essential thrombocythemia (ET), and primary myelofibrosis (PMF). In 2005, the discovery of *JAK2*V617F mutation created a breakthrough in the diagnosis of *BCR-ABL1*-negative MPNs (Campbell et al., 2005; James et al., 2005; Kralovics et al., 2005). The *JAK2*V617F was present in roughly 90% of patients with PV and in 50% to 60% of those with ET or PMF. In addition, *MPL* exon 10 mutations (mainly involving codon W515) were found in 5% to 10% of patients with *JAK2*V617F-negative ET and PMF (Pardanani et al., 2006; Pikman et al., 2006). Recently, novel frameshift mutations in exon 9 of the calreticulin (*CALR*) gene were identified in ET or PMF patients without *JAK2* and *MPL* mutations (Nangalia et al., 2013). Approximately 70 different indels in *CALR* exon 9 were classified into *CALR* type 1 (c.1099_1150del; L367fs*46: 50% of all types), *CALR* type 2 (c.1154_1155insTTGTC; K385fs*47: 30% of all types), and *CALR* other types (Al Assaf et al., 2015; Kim et al., 2015). The somatic mutations in *JAK2*, *CALR*, and *MPL* were included in the World Health Organization (WHO) classification of MPNs (Arber et al., 2016). Several studies have shown that *JAK2*V617F-mutated ET patients had older age, lower platelet counts, higher hemoglobin levels, higher leukocyte counts, and higher thrombotic risk compared with *CALR*-mutated cases (Al Assaf et al., 2015; Cazzola and Kralovics, 2014; Tefferi et al., 2014). However, *CALR*-mutated ET had a relatively higher risk of myelofibrotic transformation, especially in cases with *CALR* type 1 mutation (Pietra et al., 2016).

To the best of our knowledge, the characteristics of Vietnamese patients with ET remains mostly undetermined. In this study, we investigated the profiles of *JAK2*V617F, *MPL*, and *CALR* mutations in Vietnamese ET patients using allele specific oligonucleotide – polymerase chain reaction (ASO-PCR) and conventional Sanger sequencing method. The clinical and hematological features were compared according to mutation patterns.

## Materials and Methods

*Patients and samples*

This was a retrospective study of 395 patients diagnosed with ET between 2008 and 2017 at Blood Transfusion and Hematology Hospital at Ho Chi Minh City, Vietnam. The diagnosis of ET was established based on the 2008 WHO diagnostic criteria (Campo et al., 2011). In brief, patient was diagnosed with ET when he/she had thrombocytosis, megakaryocyte proliferation, and did not meet WHO criteria for other MPNs, myelodysplastic syndrome (MDS) or myeloid neoplasm. Clinical and hematological findings at diagnosis were obtained by reviewing the medical records. Written informed consents for mutation analyses were obtained from patients enrolled in this study. Genomic DNA was extracted from peripheral blood samples using the GeneJET Genomic DNA Purification Kit (Thermo Scientific, Waltham, MA, USA) according to the manufacturer’s instruction.

*Mutation analysis*

All primers used in this study were newly designed. All 395 samples were assessed for *JAK2*V617F status using ASO-PCR technique. Genomic DNA was amplified in a 35-cycle PCR reaction at an annealing temperature of 60^o^C using three primers. The reaction contained 25 – 50 ng of genomic DNA, 1X PCR Buffer, 200 µM each dNTP, 0.5 U Taq Hot Start Polymerase (Takara Bio, Shiga, Japan), 0.2 µM common forward primer, 0.1 µM each of reverse primers. The mutant allele showed two bands at 453 base pairs (bp) and 279 bp, while the wild-type allele had only one band at 453 bp. Primers were as follows: reverse wild-type – specific primer, 5’-attgctttcctttttcacaagat-3’; reverse mutant – specific primer, 5’-gttttacttactctcgtctccacaaaa-3’; and common forward primer, 5’-tcctcagaacgttgatggcag-3’.

Patients with non-mutated *JAK2*V617F were further evaluated for *CALR* exon 9 and *MPL* exon 10 mutations using Sanger sequencing method. The *CALR* exon 9 was amplified with primers *CALR*-F (5’-gaaaccctgtccaaagcaag -3’) and *CALR*-R (5’-agagacattatttggcgcgg-3’); while *MPL* exon 10 was amplified with primers *MPL*-F (5’-tttgggtcaaacagacgctg-3’) and *MPL*-R (5’-cacagagcgaaccaagaatg-3). Each reaction consists of 1X PCR Buffer, 1.5 mM MgCl2, 200 µM each dNTP, 0.5 U Taq Hot Start Polymerase (Takara Bio), 0.1 µM each forward and reverse primers, and 25 – 50 ng of genomic DNA. PCR involved an initial denaturation at 98°C for 3 min followed by 40 cycles of 98°C for 10 sec, 60°C for 30 sec, and 72°C for 1 min with a final elongation of 72°C for 5 min. PCR products were checked for size and purity using 1.5% agarose gel electrophoresis. PCR products were purified enzymatically using ExoSAP IT™ PCR Product Cleanup Reagent (Thermo Scientific) for removal of excess primers and dNTPs prior to Sanger sequencing using a BigDye Terminator v3.1 Kit and ABI 3500 Genetic Analyzer (Applied Biosystems, Foster City, CA, USA). PCR fragments were sequenced and analyzed in both directions.

*Statistical analysis*

The clinical and hematological findings were summarized by each of the four groups of mutational status (*JAK2*, *CALR*, *MPL*, and triple-negative) and were compared between each pair of these groups using two-sided Fisher’s exact test for categorical variables and Mann-Whitney U test for numeric variables, where appropriate. The thrombotic-event-free survival rate was described by mutational status using Kaplan-Meier estimate. Statistical significance was defined as P-value less than 0.05. All statistical analyses were performed using the statistical software R version 3.4.4. 

## Results

*Baseline clinical characteristics and prevalence of mutation*

Among 395 patients diagnosed with ET, the follow-up duration ranged from 1 to 13 years, with the median length of follow-up of 3 years. The baseline clinical characteristics are shown in Table 1. There were more females than males (249/146). The median age was 54 years and more than 75% of the patients were middle-aged or older. There were 34 patients (8.6%) with history of arterial thrombotic diseases. According to the IPSET-thrombosis risk score, 130 patients (32.9%) had high risk and 112 patients (28.4%) had intermediate risk of thrombosis. The laboratory data showed normal median values of red blood cell (RBC) counts, hemoglobin (HGB) concentration, and white blood cell (WBC) counts. The median platelet count was 1037 × 10^9^/L. There were also high values of megakaryocytes, lactate dehydrogenase (LDH), and serum uric acid.

Two hundred twenty-two patients (56.2%), 109 patients (27.6%), and 4 patients (1%) harbored *JAK2*V617F mutation, *CALR* mutations, and *MPL* mutations, respectively; leaving 60 patients (15.2%) negative for all three mutational tests. Of 109 *CALR*-mutated patients, *CALR* type 1 mutation (c.1099_1150del) was the most common, accounting for 61 patients (56%). Thirty-six patients (33%) carried *CALR* type 2 mutation (c.1154_1155insTTGTC). In the remaining 12 cases (11%), ten types of *CALR* mutations were detected as shown in Table 2. Four different types of *MPL* exon 10 mutations detected were* S505N*, *W515K*, *W515L*, and *W515S*.

**Table 1:** Baseline Characteristics and Prevalence of Mutations in Patients with Essential Thrombocythemia

Characteristics	Summary statistics
Number of patients, n	395
Male, n (%)	146 (37.0)
Age (years), median (IQR)	54 (41, 66)
Comorbidities, n (%)	
- Hypertension	85 (21.5)
- Dislipidemia	38 (9.6)
- Arterial thrombosis history	34 (8.6)
- Diabetes	19 (4.8)
IPSET-thrombosis risk, n (%)	
- Low	153 (38.7)
- Intermediate	112 (28.4)
- High	130 (32.9)
Laboratory data, median (IQR)	
- RBC, ´ 10^12^/L	4.5 (4.0, 4.9)
- HGB, g/dL	12.5 (11.2, 13.9)
- WBC, ´ 10^9^/L	12.1 (9.6, 16.4)
- Platelets, ´ 10^9^/L	1037 (793, 1342)
- Megakaryocyte^a^	80 (50, 100)
- LDH, IU/L^b^	258 (217, 363)
- Acid uric, mg/dL^c^	318 (260, 385)
Mutation profiles, n (%)	
- JAK2	222 (56.2)
- CALR	109 (27.6)
CALR type 1	61 (15.4)
CALR type 2	36 (9.1)
CALR other types	12 (3.0)
- MPL	4 (1.0)
- Triple-negative	60 (15.2)

^a^, The number of patients was 315; ^b^, The number of patients was 302; ^c^, The number of patients was 362; HGB, hemoglobin; IPSET, International Prognostic Score for Thrombosis in Essential Thrombocythemia; IQR, interquartile range; LDH, lactate dehydrogenase; RBC, red blood cell; WBC, white blood cell.

**Table 2:** Mutational Characteristics of Patients with CALR Mutation

*CALR* mutation type		n	%
c.1099_1150del	p.L367fs*46	61	56
c.1154_1155insTTGTC	p.K385fs*47	36	33
c.1100_1145del	p.L367fs*48	3	11
c.1105_1138del	p.E369fs*50	1	
c.1121_1139del	p.K374fs*50	1	
c.1124_1142del	p.K375fs*49	1	
c.1147_1151>TGGT	p.E383fs*47	1	
c.1149_1150insCAGAG	p.D384fs*48	1	
c.1149_1154>TCCTTGTC	p.E383fs*48	1	
c.1153delA	p.K385fs*45	1	
c.1116_1146del	p.D373fs*47	1	
c.1129_1140>CTTTGCGA	p.K377fs*52	1	
Total		109	100

**Figure 1:**
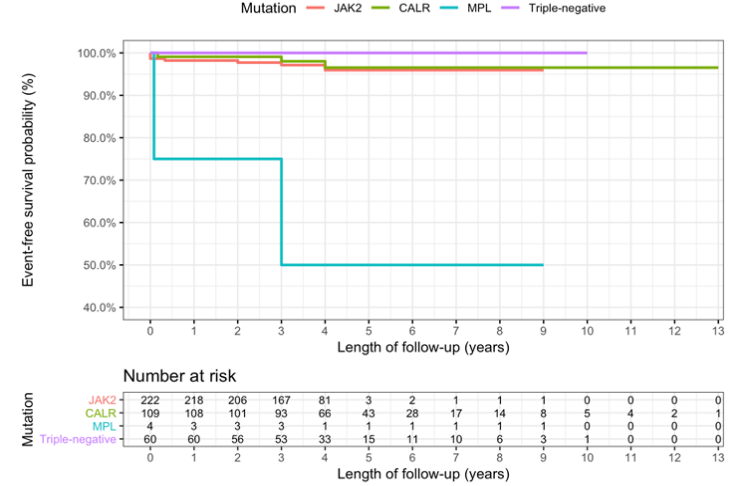
Kaplan-Meier Curves for Thrombotic-event-free Survival by Mutational Status

**Table 3:** Clinical and Laboratory Features Stratified by Mutational Status

Characteristics	*JAK2* ^(1)^	*CALR* ^(2)^	*MPL* ^(3)^	Triple-negative^(4)^	*P* ^1vs.2^	*P* ^1vs.3^	*P* ^1vs.4^	*P* ^2vs.3^	*P* ^2vs.4^	*P* ^3vs.4^
Number of patients, n	222	109	4	60						
Male, n (%)	87 (39.2)	35 (32.1)	3 (75.0)	21 (35.0)	0.227	0.304	0.654	0.110	0.735	0.144
Age, years	57 (45, 68)	51 (41, 61)	71 (56, 77)	45 (32, 59)	0.005	0.379	<0.001	0.186	0.065	0.138
IPSET-thrombosis risk group, n (%)					<0.001	<0.001	<0.001	0.096	0.630	0.056
- Low	1 (0.5)	95 (87.2)	2 (50.0)	55 (91.7)						
- Intermediate	98 (44.1)	10 (9.2)	1 (25.0)	3 (5.0)						
- High	123 (55.4)	4 (3.7)	1 (25.0)	2 (3.3)						
RBC, ´ 10^12^/L	4.8 (4.3, 5.3)	4.2 (3.9, 4.5)	4.4 (3.8, 4.9)	4.2 (3.7, 4.5)	<0.001	0.282	<0.001	0.756	0.507	0.657
HGB, g/dL	13.2 (11.8, 14.4)	11.8 (10.8, 12.8)	11.2 (10.2, 12.4)	11.5 (10.0, 12.6)	<0.001	0.101	<0.001	0.603	0.112	0.989
WBC, ´ 10^9^/L	14.0 (10.8, 18.4)	9.7 (8.1, 11.9)	6.3 (5.3, 7.5)	11.3 (9.6, 13.8)	<0.001	0.001	<0.001	0.014	0.017	0.007
Platelets, ´ 10^9^/L	950 (755, 1178)	1207 (900, 1477)	1111 (938, 1318)	1153 (998, 1453)	<0.001	0.291	<0.001	0.852	0.948	0.760
Megakaryocyte	80 (50, 100)	60 (30, 100)	30 (30, 30)	80 (58, 100)	0.015	0.200	0.391	0.370	0.011	0.162
LDH, IU/L	273 (224, 352)	263 (223, 376)	230 (197, 434)	249 (190, 398)	0.796	0.679	0.250	0.579	0.189	0.856
Acid uric, mg/dL	335 (279, 419)	296 (242, 352)	341 (294, 373)	302 (242, 364)	<0.001	0.804	0.030	0.510	0.689	0.721
Hepatomegaly, n (%)	10 (4.5)	4 (3.7)	0 (0)	3 (5.0)	1	1	1	1	0.700	1
Splenomegaly, n (%)	29 (13.1)	7 (6.4)	0 (0)	4 (6.7)	0.090	1	0.256	1	1	1
Thrombotic events, n (%)	6 (2.7)	2 (1.8)	2 (50.0)	0 (0)	1	0.006	0.348	0.006	0.539	0.003
Mortality, n (%)	1 (0.5)	1 (0.9)	0 (0)	0 (0)	0.551	1	1	1	1	-

Summary statistic is absolute count (%) for categorical variables and median (IQR) for continuous data; P values were calculated between patients in each pair of mutations. Significance values are in boldface; HGB, hemoglobin; IPSET, International Prognostic Score for Thrombosis in Essential Thrombocythemia; IQR, interquartile range; LDH, lactate dehydrogenase; RBC,red blood cell; WBC, white blood cell.

**Table 4:** Clinical and Laboratory Features Stratified by CALR Mutation Subtypes

Characteristics	*CALR* type 1 ^(1)^	*CALR* type 2^ (2)^	*CALR* other types ^(3)^	*P* ^1vs.2^	*P* ^1vs.3^	*P* ^2vs.3^
Number of patients, n	61	36	12			
Male, n (%)	17 (27.9)	11 (30.6)	7 (58.3)	0.819	0.051	0.101
Age, years	50.0 (42.0, 62.0)	50.5 (37.2, 60.2)	60.0 (56.2, 61.5)	0.976	0.052	0.045
IPSET-thrombosis risk group, n (%)				0.641	0.757	0.482
- Low	53 (86.9)	30 (83.3)	12 (100.0)			
- Intermediate	5 (8.2)	5 (13.9)	0 (0.0)			
- High	3 (4.9)	1 (2.8)	0 (0.0)			
RBC, ´ 10^12^/L	4.2 (3.9, 4.5)	4.2 (4.0, 4.4)	4.4 (4.2, 4.7)	0.991	0.368	0.289
HGB, g/dL	11.8 (10.5, 12.8)	11.7 (11.1, 12.6)	12.3 (11.9, 13.0)	0.646	0.198	0.359
WBC, ´ 10^9^/L	10.3 (8.3, 12.2)	9.3 (7.8, 11.0)	9.7 (8.8, 11.2)	0.055	0.503	0.475
Platelets, ´ 10^9^/L	1187 (928, 1473)	1277 (989, 1560)	917 (788, 1237)	0.378	0.228	0.116
Megakaryocyte	60 (30, 80)	80 (30, 100)	50.0 (30, 70)	0.391	0.258	0.095
LDH, IU/L	279 (223, 384)	250 (222, 339)	256 (232, 494)	0.655	0.917	0.725
Acid uric, mg/dL	296 (248, 354)	293 (238, 350)	313 (229, 372)	0.789	0.641	0.482
Hepatomegaly, n (%)	2 (3.3)	0 (0)	2 (16.7)	0.528	0.124	0.059
Splenomegaly, n (%)	3 (4.9)	2 (5.6)	2 (16.7)	1	0.187	0.257
Thrombotic events, n (%)	2 (3.3)	0 (0)	0 (0)	0.528	1	-
Mortality, n (%)	1 (1.7)	0 (0)	0 (0)	1	1	-

Summary statistic is absolute count (%) for categorical variables and median (IQR) for continuous data; P values were calculated between patients in each pair of mutations. Significance values are in boldface. HGB, hemoglobin; IPSET, International Prognostic Score for Thrombosis in Essential Thrombocythemia; IQR, interquartile range; LDH, lactate dehydrogenase; RBC, red blood cell; WBC, white blood cell.

*Clinical characteristics with different mutational status*

Clinical characteristics by mutational groups are shown in Table 3.There was no significant difference in sex ratio between groups. Compared to *JAK2*-mutated patients (*JAK2* group), *CALR*-mutated (*CALR* group) and triple-negative patients (triple-negative group) were significantly younger (*JAK2* group: 57 years; *CALR* group: 51 years; and triple-negative group: 45 years), had lower risk of thrombotic events based on IPSET-thrombosis risk score, showed lower RBC counts (*JAK2* group: 4.8 × 10^12^/L; *CALR* group: 4.2 × 10^12^/L; and triple-negative group: 4.2 × 10^12^/L), lower HGB levels (*JAK2* group: 13.2 g/dL; *CALR* group: 11.8 g/dL; and triple-negative group: 11.5 g/dL), lower WBC counts (*JAK2* group: 14 × 10^9^/L; *CALR* group: 9.7 × 10^9^/L; and triple-negative group: 11.3 × 10^9^/L), higher platelet counts (*JAK2* group: 950 × 10^9^/L; *CALR* group: 1207 × 10^9^/L; and triple-negative group: 1153 × 10^9^/L), and lower serum uric acid concentration (*JAK2* group: 335 mg/dL; *CALR* group: 296 mg/dL; and triple-negative group: 302 mg/dL). No significant difference was observed concerning hepatomegaly, splenomegaly, and thrombotic events between these three groups. Two patients died during follow-up period. Kaplan-Meier estimates for thrombotic-event-free survival by mutational status were shown in Figure 1. Among four cases with *MPL* mutation, two experienced thrombotic events with no mortality. There was no significant difference among *JAK2*, *CALR*, and triple-negative groups regarding the event-free survival estimate.

*Clinical characteristics with different CALR mutation subtypes*

The clinical characteristics by *CALR* mutation subtypes are shown in Table 4. While patients with *CALR* types 1 and types 2 were younger than other *CALR* types, there was no significant difference concerning sex ratio and IPSET-thrombosis risk score between these subtypes. Compared with *CALR* type 1, *CALR* type 2 patients seemed to have lower WBC counts, higher platelet counts, and lower LDH concentration; however, the differences were not statistically significant. Hepatomegaly, splenomegaly, thrombotic events, and mortality were rare in all subtypes.

## Discussion

This is the first comprehensive study to describe the profile of Vietnamese patients with ET. Using ASO-PCR and conventional Sanger sequencing method, we found that 84.8% of 395 ET patients carried *JAK2*V617F, *CALR*, or *MPL* mutations, underscoring the importance of combined genetic tests for diagnosis of ET patients. The *JAK2*V617F was the most frequent mutation (56.2%), followed by *CALR* mutation (27.6%), which is consistent with Chinese (Lin et al., 2015), Japanese (Misawa et al., 2018), Argentinean (Ojeda et al., 2018), and American (Tefferi et al., 2014) ET populations. However, the frequency of *CALR* exon 9 mutations in our study is higher than that of Thai (Limsuwanachot et al., 2017), Korean (Kim et al., 2015) , Italian (Rotunno et al., 2014) , Polish (Wojtaszewska et al., 2015) , and Brazilian (Nunes et al., 2015) ET patients; in those populations the mutation rate of *CALR* exon 9 mutations ranged from 12.5% to 15.5%. Twelve different types of *CALR* mutation including deletion, insertion and complex indels were found in our study. Type 1 (c.1099_1150del), type 2 (c.1154_1155insTTGTC), and other indels were detected in 56%, 33%, and 11%, respectively, in good agreement with previous reports (Al Assaf et al., 2015; Klampfl et al., 2013; Nangalia et al., 2013; Tefferi et al., 2014).

Among four different types of *MPL* exon 10 mutations detected (S505N, W515K, W515L and W515S), S505N was reported as a founder mutation in several pedigrees with familial thrombocytosis (Ding et al., 2004). However, it was also found as an acquired somatic mutation in rare ET patients (Beer et al., 2008; Vainchenker and Kralovics, 2017). In this study, the patient carrying *MPL*S505N was an 80-year-old man with HGB level of 10.2 g/dL, platelet count of 1,421 x 10^9^/L, and WBC count of 5.71 x 10^9^/L at diagnosis. He had history of thrombotic event and developed secondary bone marrow fibrosis three years after diagnosis.

Among *JAK2*V617, *CALR* and triple-negative groups, we showed here that triple-negative ET patients were the youngest, quite similar to previous studies (Al Assaf et al., 2015; Ojeda et al., 2018). Also, consistent with previous reports, patients with *JAK2* mutations had significantly higher HGB level, higher RBC and WBC counts, and higher thrombosis risk score, but lower platelet counts compared with patients with *CALR* mutations or triple-negative for mutations (Al Assaf et al., 2015; Ojeda et al., 2018; Rumi et al., 2014; Tefferi et al., 2014).

When comparing the clinical and hematological findings among *CALR* type 1, *CALR* type 2, and other *CALR* types, we found that three *CALR* mutation groups were similar in their sex ratio, IPSET-thrombosis risk score, HGB level and WBC counts. In a similar cohort study of 402 ET patients, Tefferi et al concluded that patients with *CALR* type 2 had significantly higher platelet count compared with *CALR* type 1 (Tefferi et al., 2014). In our study, patients with *CALR* type 2 showed a tendency of higher platelet counts; however, there was no significant difference among these three *CALR* mutation groups (Table 3).

Within a 3-year median time of follow-up, thrombotic events and mortality were rare, with two death cases and ten thrombotic events. Long-term follow-up is required to further explore thrombotic events and mortality rate based on mutational groups. A limitation of our study is that this was a single-center retrospective study with a limited sample size. Therefore, only four patients harbored *MPL* mutations detected, with two of them experiencing thrombotic events. Although the prevalence of thrombotic events in the *MPL* mutation group was higher than in other groups, we could not conclude that this group was associated with worse outcome due to small number of patients.

In conclusion, this study is the first comprehensive investigation of gene mutations in Vietnamese patients with ET. The combined genetic tests can clarify approximately 85% of the ET patients with *JAK2*V617F, *CALR* exon 9, and *MPL* exon 10 mutations, which might improve the diagnosis and classification of ET in Vietnam.
